# Electrospun PVDF Nanofibers for Piezoelectric Applications: A Review of the Influence of Electrospinning Parameters on the β Phase and Crystallinity Enhancement

**DOI:** 10.3390/polym13020174

**Published:** 2021-01-06

**Authors:** Zhongchen He, François Rault, Maryline Lewandowski, Elham Mohsenzadeh, Fabien Salaün

**Affiliations:** 1 ENSAIT, GEMTEX—Laboratoire de Génie et Matériaux Textiles, F-59000 Lille, France; zhongchen.he@ensait.fr (Z.H.); francois.rault@ensait.fr (F.R.); maryline.lewandowski@ensait.fr (M.L.); elham.mohsenzadeh@junia.com (E.M.); 2 Junia, F-59000 Lille, France; 3 Univ. Lille, F-59000 Lille, France

**Keywords:** electrospinning, polyvinylidene fluoride, piezoelectric properties, crystallinity, β phase

## Abstract

Polyvinylidene fluoride (PVDF) is among the most attractive piezo-polymers due to its excellent piezoelectricity, lightweight, flexibility, high thermal stability, and chemical resistance. PVDF can exist under different forms of films, membranes, and (nano)fibers, and its piezoelectric property related to its β phase content makes it interesting for energy harvesters and wearable applications. Research investigation shows that PVDF in the form of nanofibers prepared by electrospinning has more flexibility and better air permeability, which make them more suitable for these types of applications. Electrospinning is an efficient technique that produces PVDF nanofibers with a high β phase fraction and crystallinity by aligning molecular dipoles (–CH2 and –CF2) along an applied voltage direction. Different nanofibers production techniques and more precisely the electrospinning method for producing PVDF nanofibers with optimal electrospinning parameters are the key focuses of this paper. This review article highlights recent studies to summarize the influence of electrospinning parameters such as process (voltage, distance, flow rate, and collector), solution (Mw, concentration, and solvent), and ambient (humidity and temperature) parameters to enhance the piezoelectric properties of PVDF nanofibers. In addition, recent development regarding the effect of adding nanoparticles in the structure of nanofibers on the improvement of the β phase is reviewed. Finally, different methods of measuring piezoelectric properties of PVDF nanofibrous membrane are discussed.

## 1. Introduction

Piezoelectric materials convert mechanical energy into electrical energy or vice versa according to direct or inverse piezoelectric effects [1,2] and, as such, find numerous applications in electromechanical fields [3,4,5,6,7,8,9,10]. Among these materials, piezoelectric polymers have attracted significant attention in many research fields due to their intrinsic properties (processability, flexibility, etc.) compared to ceramics and other kinds of materials. Fluorinated polymers such as polyvinylidene fluoride (PVDF) and its copolymers are particularly studied for the development of polymeric piezoelectric devices. PVDF has a broad variety of properties including sufficient availability for mass applications and a contained price (e.g., around 20 €/kg vs. 1000 €/kg for PVDF-TrFE). PVDF also comes with a high purity, and it is lightweight and flexible, resistant to solvents and stable under high electric fields [3]. This explains its more extensive use compared to that of other copolymers with higher piezoelectric constants. PVDF ((–CH_2_–CF_2_–)_n_) is a semicrystalline polymer, obtained from the free-radical polymerization of CH_2_=CF_2_. The PVDF crystalline structure exhibits five different polymorphs, i.e., α, β, γ, δ, and ε phases, depending on crystallization conditions [11]. The nonpolar α phase and the polar β and γ phases have been the most studied, as they constitute the most significant proportion in the PVDF crystalline structure. The α phase is the common form and the most thermodynamically stable phase, and the β phase shows the best piezoelectric responses with the highest dipolar moment per unit cell (8 × 10^−30^ C·m) [12,13]. Thus, the piezoelectric performance of PVDF is mostly related to the presence of polar crystalline phases and in particular the β phase [14]. It is because of its potentially high electroactive properties that PVDF possesses under different forms of films, membranes, and fibers, and therefore, PVDF is widely used in energy harvesters [4], sensors, actuators [15,16,17,18], battery separators [19,20,21,22], and biomedical applications [23,24,25]. Furthermore, PVDF nanofibers are mainly used for energy harvesting and battery separators applications [26,27,28]. The copolymerization of PVDF with TrFE, i.e., –(CHF–CF_2_)–, and tetrafluoroethylene (TeFE; –(CF_2_–CF_2_)–) in a random sequence allows for the improved formation of the β phase by copolymerization without the use of additional post-treatments. The presence of fluorine atoms induces additional steric hindrance, thus limiting the formation of the α phase. In addition, the degree of crystallinity and alignment of the CF_2_ dipoles produce higher piezoelectric and pyroelectric effects than those of PVDF as a result of annealing, mechanical stretching, or electrical poling treatments. The structural and piezoelectric properties of P(VDF-TrFE) are strongly dependent on the TrFE content [29].

The piezoelectric performance of PVDF is related to its β phase structure and its degree of crystallization. The relative ratio of the β phase content in PVDF is usually measured by Equation (1):

(1) $$F\left(\beta \right)=\frac{X_{\beta }}{X_{\beta }+X_{\alpha }}\times 100\%=\frac{A_{\beta }}{\frac{K_{\beta }}{K_{\alpha }}A_{\alpha }+A_{\beta }}\times 100\%,$$

where X_α_ and X_β_ are the degrees of the PVDF α and β phases; K_α_ (6.1 × 10^4^ cm^2^·mol^−1^) and K_β_ (7.7 × 10^4^ cm^2^·mol^−1^) are the absorption coefficients at the at 766 and 840 cm^−1^; A_α_ and A_β_ are the absorption bands at 766 and 840 cm^−1^, respectively, corresponding to the α and β phases shown in the Fourier-transform infrared (FTIR) spectra, respectively.

However, the content of the β phase in PVDF is not only related to F(β), but also related to the degree of crystallization (X_c_). The crystallinity is usually calculated based on Equation (2):

(2) $$X_{c}=\frac{△H}{△H_{m}\varphi }\times 100\%,$$

where △H is the fusion enthalpy of the PVDF samples according to the DSC curves, △H_m_ is the fusion enthalpy of PVDF with 100% crystallinity (104.7 J·g^−1^) [30], and φ is the PVDF weight fraction in PVDF nanofibers or membranes.

This paper aims to present a comprehensive review of the current state of the art in preparing PVDF nanofibers via electrospinning technology. In recent years, many researchers have been interested in the development of flexible PVDF-based nanogenerators having good piezoelectric performance. Electrospinning of PVDF, as a method of manufacturing nanofibers, is favorable for the improvement of piezoelectric performance due to the formation of the β phase and crystal orientation. Therefore, the review focuses on the effects of system parameters, such as operating and working solution parameters, on the improvement of piezoelectric properties, which are mainly attributed to their β phase content and their crystallinity. Meanwhile, some relationships among operating parameters and the formation of the nanofiber and final structure, as well as the beta phase formation, are also summarized. Finally, this review provides detailed guidance about the selection of a suitable solvent system concerning the desired morphology and structure of electrospun PVDF fibers for potential application in harvesting systems.

## 2. Production of Nanofibers

**Figure 1:**
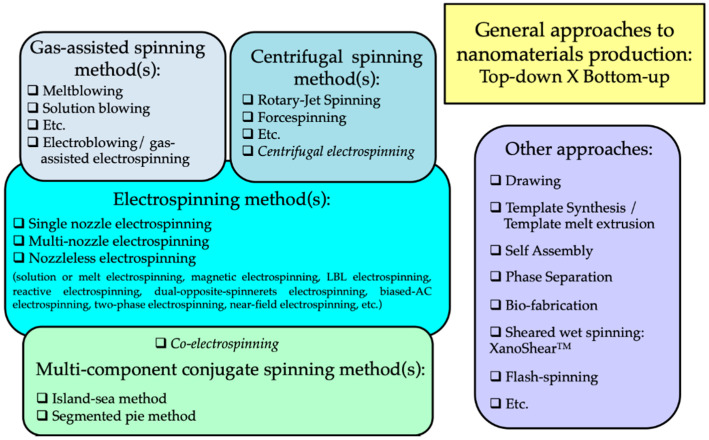
Classification of nanofibers-producing methods.

In the literature, the term “nanofiber” has various definitions. This includes fibers with diameters in the nanometer scale, nanocomposite fibers, and nanostructured surface fibers. Nanofibers, as part of nanomaterials, have at least one dimension equal or less than 100 nanometers. They also have a length-to-diameter ratio higher than 100 (L/D > 100). Although the term “nanofiber” is used for fibers with a mean diameter lower than 100 nm according to the International Standards Organization (ISO) [31], it is also accepted for fibers with a diameter lower than 1000 nm in the engineered fibers industries [32]. The decrease in the size of materials and their entry into the field of nanomaterials allows the development of materials with unique and improved properties or functionalities compared to the same materials at a macroscale. Considering fibers, a reduction of diameter leads principally to an increase of specific surface area. A high surface area of nanofibers provides modifications on bioactivity (e.g., increase of the cell proliferation), electroactivity, strength, etc. The applications of nanofibers cover various aspects, such as filter materials [33,34,35,36,37,38,39], electronic technology (e.g., nanoelectronics, optical sensors, battery electrode materials, and capacitors) [40,41,42,43,44,45], biomedical science (e.g., such as tissue engineering and drug delivery) [46,47,48,49], and catalyst supports [50,51]. There are different methods based on the top-down or bottom-up approaches to produce nanofibers, as shown in Figure 1.

These nanofibers-producing methods can be divided into five distinct groups. One group of them includes methods using gases to draw the produced fibers to reach the desired diameters. It is commonly called gas-assisted spinning methods, including meltblowing and solution blowing [52]. A second one uses a centrifugal force to prepare nanofibers, called centrifugal spinning methods including rotary-jet spinning and forcespinning [53,54]. A third group is based on the use of traditional spinning methods that simultaneously employ two or three different polymers (modified or not) and spinnerets with specific geometries. The various elements produced by these multicomponent conjugants spinning methods are finally dissociated after a subsequent operation (dissolution or splitting process) to obtain nanofibers. A fourth group is dedicated to the methods using an electric field for the development of nanofibers [55]. They can be described as electrospinning methods. They refer to the solution or melt electrospinning methods using single, multiple, or no nozzle. At the interface between this fourth group and the three previous ones, methods combining two aspects appear, for example, the centrifugal electrospinning which uses an electric field and a centrifugal force to draw a fiber into the nanometer size. Electrospinning with no nozzle, called needleless electrospinning, is developed to improve the productivity rate of nanofibers and to upscale obtained results on a needle electrospinning device. Nanospider™ needle-free technology presented by Elmarco Company is a good example for this purpose. In addition to all these methods, there are some others such as drawing, template synthesis, self-assembly, phase separation, and flash spinning [55,56,57,58,59], but most of them are only limited to the laboratory scale.

The main techniques used to produce PVDF nanofibers are the conventional solution or melt electrospinning [60,61,62,63] and the alternative centrifugal spinning [3]. In this latter method, a polymer or polymer solution is expelled from spinnerets by a centrifugal force and a shear force during the high-speed rotation of a device. Compared with other technologies mentioned above, electrospinning has many irreplaceable advantages. First of all, electrospinning comprises two methods, solution and melt electrospinning allowing covering a wide range of polymers. However, nanofibers are mainly prepared by solution electrospinning. The electrospinning process also provides effective control over the characteristics of PVDF nanofibers that govern their piezoelectric properties. Some articles reported that the β phase content in electrospun PVDF nanofibers is higher than those obtained by other methods [64,65]. During the electrospinning process, the flow of the polymer under an electric field induces the orientation of the polymer chain and polarizes the fibers. This can effectively improve the crystallinity and the β phase of PVDF [66], which cannot be achieved in one step by melt electrospinning or centrifugal spinning [67,68]. Thus, due to the advantages of solution electrospinning in the preparation of piezoelectric nanofibers, the development of PVDF nanofibers by this technique will be especially described in this review.

## 3. Experimental Setup and Process Parameters of Electrospinning

Solution and melt electrospinning are two very simple processes in principle: an electrical potential is applied between a polymer, more particularly between the physical zone of jet emission (i.e., the spinneret) or its substitute (in the case of needleless electrospinning), and an electrically conductive surface acting as a collector for the produced fibers. The electric field generated by a high applied voltage stretches the fluid which is forced to take a very particular shape called Taylor cone. When the electrical voltage is higher than a critical value, the surface tension is no longer sufficient to maintain the cone and the polymer jet is initiated and drawn to the collector. In melt electrospinning, a temperature above the melting point of the polymer is applied to ensure its flow through the nozzle, and the fiber is obtained after elongational stretching during the flying time. This process is mainly used, when it is difficult to find a proper solvent at room temperature for the polymer. Even if the process has a high efficiency, the mean diameter of the fibers obtained is usually higher than those obtained by a solution electrospinning process. Furthermore, such a process requires specific equipment and much higher voltages due to the low electrical conductivity and high viscosity of molten polymers.

**Figure 2:**
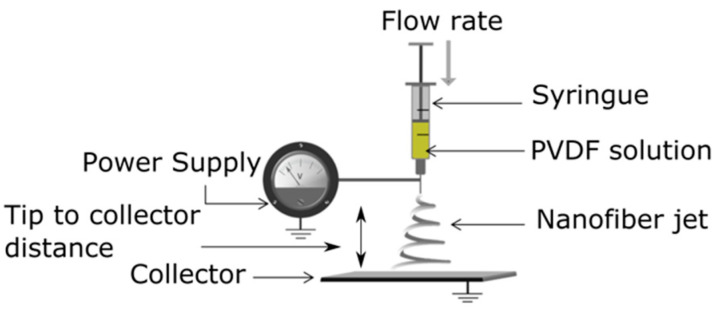
Schematic diagram of an electrospinning setup.

The basic principle of electrospinning consists of the uniaxial stretching of a viscoelastic polymer solution in an electrical field. The schematic principle of a solution electrospinning setup is shown in Figure 2. It consists of a syringe pump equipped with a needle (tip extrusion system), a high voltage power supply, and an electrically conducting collector.

A syringe is filled with a polymer solution, a syringe pump is set at a certain feed rate, and a proper voltage is applied between the needle and the collector. The process parameters are adjusted to ensure the formation of a Taylor cone at the tip of the needle. The fine jet extrudes from the Taylor cone, when the electrostatic forces are higher than the surface tension and the viscous forces. The solvent evaporation during the flying process allows the formation of solid nanofibers recovered on the collector surface.

**Figure 3:**
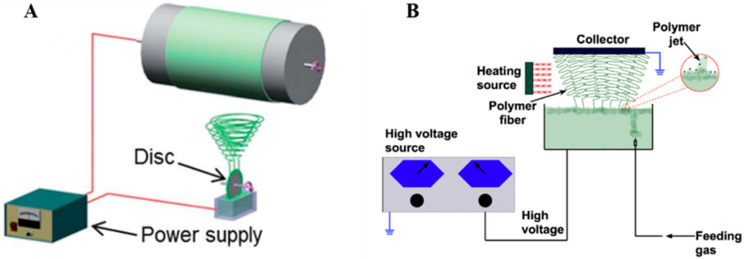
(**A**) Schematic diagram of needleless electrospinning [69]. (**B**) Bubble-electrospinning [71].

To overcome certain drawbacks, and in particular the low productivity of PVDF electrospinning, a great deal of attention has been paid, in recent years, to the modification of the process and device, namely needleless techniques. In one technique proposed by Fang et al. [69] (Figure 3A), a rotary aluminum disc is partially immersed in a PVDF solution, and the solution is connected to a high-positive-voltage DC power supply. A grounded drum collector is placed at a precise distance from the disc. This device allows them to increase the productivity up to 16.9 g/h, compared to that of a needle system of 0.16 g/h, even if the piezoelectric performance of PVDF nanofibers is not significantly increased. Hernández-Navarro et al. [70] used a bubble-electrospinning technique to prepare PVDF nanofibers, which not only significantly increases the piezoelectric properties of PVDF nanofibers, but also greatly improves productivity. In the bubble-electrospinning, a grounded aluminum plate collector is set above the PVDF solution surface. Feeding gas enters the solution and promotes the solution to form a Taylor cone (Figure 3B). In this experiment, by adjusting the applied voltage, the fraction of the β phase is almost 100%.

Regardless of the traditional solution electrospinning techniques or the novel electrospinning processes described above, parameters that influence the characteristics and properties of nanofibers produced can be divided in three categories: (i) the polymer solution characteristics, (ii) the electrospinning process parameters, and (iii) the ambient parameters. Concerning the polymer solution properties, there are three main influential factors: surface tension, conductivity, and viscosity. The surface tension and conductivity are affected by the solvent, while the viscosity mainly depends on the molecular weight and the concentration of PVDF. Therefore, choosing an appropriate solvent and a proper concentration of PVDF to prepare the solution is crucial in the electrospinning technique. The molecular weight of PVDF cannot be too high, otherwise, the PVDF solution is too viscous, which is harmful to the electrospinning process [72]. The increase in viscosity leads to an increase in the time between the flow of the polymer solution from the tip to the collector, the solvent has sufficient time to evaporate, and the molecular chains have sufficient time to stretch before solidification, resulting in an increase in F(β). However, extremely high viscosity makes the solution difficult to stretch. Therefore, it is necessary to select PVDF with suitable molecular weight and concentration for the electrospinning experiment.

Under the premise of preparing a suitable PVDF solution, the electrospinning process can be controlled by adjusting other parameters such as applied voltage, feed rate, tip-to-collector distance (TCD), different types of collector, humidity, and temperature, which also affect the fibers structure and the β phase content [73,74,75]. Environmental factors such as relative humidity (RH) and temperature also affect nanofibers deposition [76,77].

## 4. Effect of Electrospinning Parameters on the β Phase and the Crystallinity of PVDF Nanofibers

### 4.1. Process Parameters

#### 4.1.1. Voltage

During the electrospinning process, the electric field has a major effect on nanofibers structure and morphology [78]. The average fiber diameter decreases with increasing applied voltage to a critical value due to the increased electrostatic forces of the polymer solution and drawing stress. Nevertheless, higher voltage values induce beaded fibers initially, until the fiber breaks, due to the increase in the drawing stress. Therefore, determining optimal values for the Taylor cone generation and controlling fiber formation is a challenge. The stretching of a polymer jet under high applied voltages during the electrospinning process orients the PVDF molecular chains dipoles, allowing the α-to-β crystalline phase changes [79], corresponding to the formation of the trans conformation required for the β phase.

In the work of Damaraju et al. [78], an increase from 12 to 25 kV induces an increase in F(β) from 67.8% to 72.4%, whereas the overall crystallinity index of PVDF is close to 53% for each voltage value. The evolution in F(β) is also accompanied by an evolution of the diameter of the fibers, with a decrease in their average diameter from 295 to 177 nm. A higher applied voltage can produce a higher charge density on the surface of the jet, thus increasing the jet velocity and imposing a higher elongation force to the jet. Therefore, the final fiber diameter gradually becomes smaller, as the applied voltage increases. The use of high voltage values allows the alignment of the electric dipoles in the PVDF solution during the flying step of the electrospinning process. There is a proportional relationship between the applied voltage and the alignment degree. Therefore, the formation of the β phase is promoted over the α one.

In addition, depending on the experimental conditions used, there is an optimized applied voltage, and it differs from one study to another, indicating the absence of a simple proportionality to F(β). Jiyong et al. have observed that an increase of the applied voltage from 14 to 20 kV leads to an increase of the Xc value from 48% to 56.3%, corresponding to an increase of F(β) from 70% to 76% [80]. Nevertheless, further increases up to 24 kV induce both decreases of Xc and F(β), which can be related to the weakened polarization of the PVDF solution during the stretching. The increase in the applied voltage during the electrospinning process leads to an increase in the number of charges in the PVDF solution, and therefore, to further stretching of the jet. In both cases, this phenomenon allows molecular orientation and therefore an increase in the crystallinity degree of the electrospun fibers. Nevertheless, above a specific value of the applied voltage, the β phase content, as well as the crystallinity of PVDF, decreases. These phenomena are related to a reduction of the flying time, which cannot allow the macromolecular polymer chains to reorganize. Thus, the β phase content increases, until an optimum electrospinning voltage is reached, and above this optimum, it is reduced due to the effect of the acceleration during the flying time imposed by the electrical field.

#### 4.1.2. TCD

The TCD mainly affects the evaporation of a solvent in a solution and the stretching of a PVDF molecular chain, but it also causes the decrease of an electric field intensity. The TCD selected in most of the current studies is fixed at 15 cm.

Most studies have shown that the effect of TCD on the β phase of PVDF nanofibers is not a simple linear relationship. The results of some researchers showed that with the change of TCD, the F(β) and fiber diameter of PVDF nanofibers are not significantly different [81,82,83]. This is because the two effects of TCD on the formation of the β phase. On the one hand, with the increase of TCD, nanofibers have enough time to stretch, and solvents have enough time to evaporate, which is conducive to the formation of the β phase. On the other hand, with the increase of TCD, the electric field intensity decreases at the same voltage, and the jet instability increases, which is harmful for the formation of β phase [84]. The experimental results of Zheng et al. indicated that as the TCD increases from 10 to 20 cm, the absorption peak of the β phase in FTIR decreases [73].

Systematic research on TCD found that with the increase of TCD, the F(β) of PVDF nanofibers increases first and then decreases. Shao et al. have observed that when TCD is increased from 9 to 15 cm, the F(β) of PVDF nanofibers increases from approximately 77% to 85.9%, the voltage and current outputs reach the maximum levels of 1.5 V and 1.6 mA, and the average diameter of the nanofibers also decreases to 284 nm [81]. Further increasing TCD, the F(β) begins to decrease due to the decrease of electric field intensity and the increase of jet instability. The effects of TCD on F(β) with an electric field (1 kV·cm^−1^) have been also investigated by the same authors. They found that under a constant electric field, with the increase of TCD, the F(β) reaches the maximum value (86.2%) at 17 cm. The above results indicated that the electric field intensity is affected by the change of TCD, which ultimately affects F(β). Therefore, a too high or too low TCD is not conducive to obtaining the maximum F(β). The TCD used in electrospinning PVDF nanofibers range from 10 to 20 cm except for some special electrospinning methods (near-field electrospinning) [85,86,87].

#### 4.1.3. Flow Rate

The polymer solution’s flow rate affects not only the formation of a Taylor cone but also the morphology of electrospun fibers. A low flow rate leads to the presence of vacuum inside a needle, whereas for higher flow rates, the polymer tends to deposit on the edge of the tip, which perturbs the Taylor cone generation. An optimum feed rate value is required to obtain and maintain a stable Taylor cone for each applied voltage. Moreover, a steady flow is required to avoid any defect in fiber formation, especially beads.

The flow rate also has a significant effect on the degree of crystallinity and F(β). For example, some authors report that increasing the flow rate results in an increase in the average fiber diameter, due to a decrease in the stretch of an electrospinning jet. Nevertheless, beads are observed at either low or higher flow rates, inducing a loss of fiber uniformity. In the first case, it is related to an increase of the surface tension of the solution during the flying process, whereas in the second case, it is due to insufficient time to allow completing solvent evaporation.

Moreover, an increase of the flow rate induces also a rapid collection of fibers on a grounded collector and the presence of a solvent, which limits the crystallization process. Thus, Jiyong et al. have observed an increase of the crystallinity for a low flow rate and its decrease at a higher flow rate [80]. However, Ribeiro et al. have observed that the flow rate variation has no significant effect on PVDF crystallinity in a range of 0.5 to 4 mL/h [88].

The polymorphous behavior of electrospun PVDF fibers is closely related to the flow rate applied. A low flow rate leads to a higher beta phase formation due to the higher stretching of a jet, allowing the formation of the β phase nuclei. Nevertheless, some contradictory results are often pointed out in the literature. On the one hand, Zheng et al. have found that a decrease in the volume flow rate, from 4.5 to 0.3 mL/h, induces an increase of the β phase fraction [73]. Similar results were found by Jiyong et al., concluding that the feed rate and F(β) have a significant inverse relationship, since the F(β) of PVDF nanofibers decreases from 78% to 72%, with an increase of the flow rate from 0.5 to 2.0 mL/h [89]. On the other hand, Singh et al. have demonstrated the increase in the β phase due to the increase in flow rate is related to the shear force exerted by a needle on a viscous polymeric solution [90]. Furthermore, they have denoted that the F(β) increases until a critical value of the flow rate (2 mL/h) and then decreases.

#### 4.1.4. Type of Collector

During the electrospinning process, conductive collectors function as a substrate to collect the charged fibers. The type of collector mainly changes the structure and performance of the obtained nanofibers (Table 1). The collectors used in different PVDF electrospinning methods are divided into static collectors and rotating collectors. Compared with the static collector, the rotating collector provides an extra stretching force during the electrospinning process to stretch the obtained PVDF nanofibers and further increase the content of the β phase.

A grounded metal plate is normally used as a traditional static collector in the electrospinning process [91,92]. The metal plate is placed at a certain distance from a needle. When a solution is ejected from the tip of a syringe, the fibers are collected directly on the metal collector without any mechanical stretching. The F(β) values are not high enough and the PVDF nanofibers are randomly distributed, when the metal plate collector is used. Shehata et al. discussed the influence of a double-metal plate collector and a conventional metal collector on F(β) [75]. For the conventional metal collector, the F(β) of the PVDF nanofibers is 74.2%. When the double-metal plate collector is selected, F(β) increases to 86%. This phenomenon is related to the fact that with the traditional collector, the electric field is not oriented in a certain direction. In addition, with the use of the double-metal plate collector, the electric field vectors form a stretching force in the gap between the two metal bars. This is an advantage for the orientation of the PVDF fibers between the two metal bars, the regular arrangement of the fibers, and the formation of the β phase.

Besides, a common method to increase the β phase is to replace the grounded metal plate collector with a rotating collector. For the static collector, only Coulomb forces provided by an applied voltage act on the electrospinning process [101]. In addition to Coulomb forces, the rotating collector can provide a mechanical stretch force to align molecular chains. In other words, the high-speed rotating collector provides elongation forces during electrospinning and organizes lamellae to form fibers aligned along the fiber axis [102]. These facilitate the conversion of the α phase to the β phase. The nanofibers obtained with rotating collectors are regularly arranged and F(β) is high, so these types of collectors are widely researched. Among the rotating collectors, although the quality of the alignment of the rotating disk collector is better than that of the rotating drum collector, it can only get a nanofibrous membrane with a very small area [103].

Abolhasani et al. have compared the crystalline structure of PVDF nanofibers obtained by using a static collector with the one obtained by a rotating collector (2500 rpm) [104]. The β phase content of PVDF nanofibers obtained by using the static collector lies in a range of 50%–60%, and nanofibers distribute randomly. However, the F(β) by using the rotating collector is varied from 70% to 80%, and nanofibers are well aligned by using the rotating collector. From another point of view, fibers collected on the rotating drum have thinner average diameters (D < 300 nm) compared to those deposited on the static collector (D < 400 nm), and the total crystallinity values of different produced PVDF nanofibers are not changed significantly (X_c_ = 40%–50%). The results demonstrated that a drum collector can effectively alter the microstructure and the β phase of PVDF nanofibers. Lins et al. have used a rotating drum as a collector to investigate the influence of the rotating speed (50, 1000, 2000, and 3000 rpm) on the β phase and the morphology of the PVDF nanofibers [102]. The fibers processed with a low speed (50 rpm) are not aligned in the rotating direction, and the diameter of the fibers is 1.98 ± 0.5 μm. As the rotating speed increases, the fibers become low-aligned (1000 rpm), medium-aligned (2000 rpm), and high-aligned (3000 rpm). The average diameter of the fibers is slightly increased at the high speeds of the collector rotation (2000 and 3000 rpm). The results of FTIR and X-ray diffraction (XRD) indicate that as the rotating speed increases, the formation of the β phase increases. Ribeiro et al. have used a rotating drum collector to analyze the influence of rotating speed on the β phase of electrospun PVDF nanofibers (500–2000 rpm) [88]. The F(β) at high rotating speeds (750–2000 rpm) is ranged from 80% to 85%, which is significantly higher than the F(β) (45%) obtained at low rotating speeds (500 rpm), but X_c_ did not change significantly (X_c_ = 58–64%).

The above literature results underlined that the use of a rotating drum as a collector, and the increase of the rotation speed can effectively improve the fraction of the β phase. However, it does not mean that the higher the rotation speed, the better the β phase formation. Wu et al. found that as the speed increases from 0 to 1500 rpm, a greater stretching and elongation of a jet takes place during electrospinning, thereby leading to more aligned fibers, and the fraction of the β phase also increases from 84% to 84.96% [105]. Nevertheless, by increasing the collector speed to 2000 rpm, due to further stretching, fiber breakage occurs, and the fraction of the β phase decreases to 78%.

### 4.2. Solution Parameters

#### 4.2.1. PVDF Molecular Weight

The molecular weight of a polymer greatly affects the viscosity of a polymer solution. For instance, even if the concentration of a PVDF solution with a low molecular weight is increased, its viscosity is smaller than that of the PVDF solution with a higher molecular weight. Viscosity plays an important role in the process of electrospinning; therefore, it is necessary to select PVDF with a suitable molecular weight for electrospinning experiments. The more commonly used molecular weights are 275,000 and 534,000 g·mol^−1^ [73,74,78,95,99,106,107,108,109,110,111].

Magniez et al. observed that the use of low-molecular-weight PVDF gives a very low viscosity polymer solution and electrospraying happens instead of electrospinning, which results in a beaded structure, while a high-molecular-weight PVDF solution gives a fiber structure. It was also found that the PVDF nanofibers prepared by the low molecular weight polymer of Kynar 710 (Mn = 70,000–80,000 g·mol^−1^) have F(β) in a range of 65%–70%, while the high-molecular-weight Kynar 740 (Mn = 250,000 g·mol^−1^) results in an F(β) value between 85% and 87% [112].

Zaarour et al. have electrospun three PVDF polymers with molecular weights of 180,000, 275,000, and 530,000 g·mol^−1^, and the obtained nanofibers showed different F(β) values of 80.38%, 85%, and 88.84, respectively [72]. As the Mw increases, the solution’s viscosity increases, and it leads to an increase in the time between the flow of the polymer solution from the tip to the collector. Hence, the solvent has sufficient time to evaporate, and the molecular chains in the jets stretch more before solidification, increasing F(β).

Therefore, to obtain high F(β), the molecular weight can be increased, but not indefinitely, as it can block the needle’s tip. Although this problem can be solved by increasing the needle and tube diameters, the fiber diameter is increased. For example, in the experiment of Fu et al., the PVDF with a Mw of 777,000 g·mol^−1^ was tested, and a high F(β) value (93.2%) was obtained [100].

#### 4.2.2. PVDF Concentration

Too low or too high PVDF solution concentration is not proper for the process of electrospinning; thus, the PVDF concentration usually used is within a range of 10–25 wt % [109,113,114]. With the increase of PVDF concentration, F(β) increases first and decreases. At low PVDF solution concentrations, there are less entanglements between polymer molecular chains, resulting in the low viscosity of the PVDF solution, which is not conducive to the electrospinning process and will form beaded nanofibers [115]. When the concentration of PVDF in the solution increases, the entanglement between macromolecular chains increases, leading to an increase of the solution viscosity, therefore resulting in more uniform nanofibers [114]. The stretching effect of the electric field on the polymer solution increases, and F(β) increases with increasing PVDF concentration. While the concentration of PVDF in the solution increases to a critical value, the same electric field has the most significant tensile effect on the polymer solution to form a uniform nanofibrous structure and F(β) reaches the maximum value. Further increase of the concentration of PVDF will increase the viscosity of the solution, and the solution will be harder to stretch due to the more robust macromolecular chain entanglement at the same voltage. Therefore, as the concentration increases, F(β) decreases.

The experimental results of Shao et al. illustrated this point, i.e., when the concentration of PVDF increases from 16% to 19%, the beads’ presence decreases, and by increasing the polymer concentration above 20%, the beads disappear [109]. Similar trends regarding the effects of the concentration on the PVDF nanofibers morphology were demonstrated in other researches regardless of the solvent used [116,117]. The F(β) increases from 78% to 85.9% until the concentration reaches 20% and then decreases to 82.5%, further increasing the concentration to 26%.

When the polymer concentration in an electrospinning solution is reduced, a higher extensional force is imparted on the flow direction of the solution, thereby producing fibers with a smaller average diameter and making a higher F(β) value possible [118]. When the PVDF concentration reduces from 22% to 12%, the average diameter decreases from 400 to 70 nm and the F(β) increases from 81.0% to 86.6%. The increase in the concentration of PVDF would reduce F(β) under the premise of obtaining the fiber structure [119].

#### 4.2.3. Solvent

Single polar solvents, such as dimethylacetamide (DMAc), dimethyl sulfoxide (DMSO), dimethylformamide (DMF), and N-methyl-2-pyrrolidone (NMP) [107,120,121,122,123], or a binary mixture of these solvents with acetone are mainly used to dissolve PVDF [108,124,125,126,127,128,129]. Adding a small amount of acetone is conducive to the volatilization of the solvent and an increase in F(β), but too much acetone is not conducive to the formation of the β phase.

The volatility of the solvent is related to the vapor pressure. The vapor pressure of acetone is more significant than DMF, DMAc, and NMP, which causes the volatility of acetone much greater than DMF, DMAc, and NMP. If only a polar solvent is selected in the preparation process of PVDF fibers, the solvent cannot be entirely evaporated while passing the distance between the syringe tip to the collector, which leads to the beads formation. The formation of the beads is not helpful for the formation of PVDF nanofibers. The addition of volatile solvents evaporates the solvent completely during the electrospinning process and reduces the structure of the beads, which is beneficial for the increase of F(β). The experimental results of Choi et al. indicate that the addition of acetone reduces the viscosity of a PVDF solution [130], which is an advantageous in the stretch of the PVDF solution and the formation of the β phase. The addition of acetone also affects the surface tension of the solution. If the solution’s surface tension is high, the electrostatic forces provided by an applied voltage cannot overcome the surface tension of the solution and only a beaded structure will be formed. The solution cannot be effectively stretched and is not conducive to high F(β) [131]. The surface tension of acetone is significantly lower than those of several other solvents. Therefore, acetone can significantly reduce the surface tension of the mixed solvent, which is beneficial to reduce the formation of the beads. Therefore, F(β) increases with the addition of the volatile solvent.

Gee et al. have prepared different PVDF nanofibers with three different solvents of DMF/acetone, DMSO/acetone, and NMP/acetone with a solvent volume ratio of 6/4 [113]. The results showed that the nanofibers produced with DMF as a significant solvent have the highest F(β) value of 90.9% and then F(β) values of 87.4% and 81.9% with DMSO and NMP, respectively. The nanofibers produced with DMF contain smoother, more organized fibers with fewer nodes. Moreover, the authors also found out the solvent ratio (acetone fraction) statistically has more considerable contribution to β phase formation compared with flow rate, TCD, and applied voltage through the analysis of the variance [132]. Therefore, they chose a mixture of DMF/acetone as a solvent to investigate the effect of the DMF/acetone volume ratio on the PVDF nanofibers formation. The results showed that as the content of acetone increases, F(β) increases. The highest mean F(β) of 81.6% is recorded with the largest acetone percentage (DMF/acetone volume ratio = 6/4). Changing the volume ratio of DMF/acetone could affect the volatility of the mixed solvent. As acetone’s content increases, the volatility of the solvent increases, and then, the solvent evaporates faster during the electrospinning. The fast solvent evaporation during the electrospinning process using a mixture solvent with high volatility could enhance the formation of the β phase. However, there is a negative impact on forming the β phase, when the acetone fraction is higher than 50 v% [84]. The negative effect of an excessively high DMF/acetone volume ratio on the formation of the β phase was also discussed in the research of Ghafari et al. [74]. They investigated the DMF/acetone volume ratio’s influence on morphologies (percentage of beads and nanofibers diameter) and F(β) in detail. The nanofibers have a lower diameter distribution for a constant applied voltage and a constant flow rate, when the DMF content increases. The amounts of beads decrease with the decrease of the DMF/acetone volume ratio. Therefore, the complete solvent evaporation is an advantage in eliminating the beads [133]. The fraction of the β phase is considerably decreased by increasing acetone content; it is different from Gee’s study that increasing acetone content increases F(β). The solvent evaporation can be divided into three levels, i.e., low, intermediate, and high evaporation rates. The low evaporation rates result mainly in the β phase formation; while the intermediate rate is beneficial for the formation of the α and β phases, the high evaporation rate is more favorable to the formation of the α phase [61]. Adding more acetone increases the solution’s evaporation rate and leads to forming more α phase in PVDF nanofiber samples, which decreases F(β). The maximum F(β) is 75%, when the DMF/acetone volume ratio is 6/4.

The above examples show that, as the volatile solvent in the mixed solvent increases, F(β) increases first and decreases. As the volatile solvent’s content increases, the PVDF solution’s viscosity decreases and the solvent evaporates entirely, leading to a higher F(β) value. When the volatile solvent’s content reaches a critical value, the fast volatilization rate is conducive to forming the α phase, resulting in a lower F(β) value.

### 4.3. Ambient Parameters

#### 4.3.1. RH

This parameter mainly affects the volatilization of a solvent, which affects the formation of the β phase of PVDF and the surface morphology of the fiber. Zaarour et al. discussed the effect of humidity on the electrospun PVDF nanofibers’ structure and physical properties [87]. They studied the surface morphology of nanofibers produced with different RH values between 5% and 65%. They found that a low RH of 5% produces a smooth surface of the nanofibers and increasing the humidity leads to the appearance of a rougher surface. In another research by Zaarour et al., it was found that increasing RH from 2% to 62% could significantly increase F(β) from 55% to 73.06% [134]. Based on their results, high humidity is inserted into the polymer jet’s surface during electrospinning, induces the deformation of the orientation of molecular chains and produces wrinkled nanofibers. This effect leads to an increase in F(β). Szewczyk et al. also showed that the F(β) values of PVDF nanofibers are 50%–52% at a RH value of 30% and 70%–73% at a RH value of 60% [135]. In the experiment of Kong et al., as the humidity increases from 10% to 70%, the F(β) of PVDF nanofibers increases from 70% to 95% and the nanofibers surface changes from a smooth surface to a rough and wrinkled surface [136]. The water from the humid environment is absorbed into PVDF nanofibers’ jet surface, forming a thin layer on the nanofibers’ surface. This thin layer can slow the evaporation rate of the solvent and retards the solidification of nanofibers. Then, the β phase has enough time to nucleate and grow.

Therefore, compared to the dry environment, the PVDF nanofibers prepared in a more humid environment have a higher F(β). To obtain a high F(β) content, the electrospinning is usually carried out under high RH [137]. However, it should be considered that when the RH reaches a critical value, the water in the environment completely inhibits the evaporation of the solvent, which makes the electrospinning process difficult [138].

#### 4.3.2. Working Temperature

The increase in working temperature increases the volatilization rate of a solvent and reduces the surface tension and viscosity of the solution. Zheng et al. compared the F(β) of PVDF nanofibers at a working temperature range of 20 to 60 °C and found that the F(β) obtained at 20 °C is the largest and, as the temperature increases, F(β) decreases [73]. They believe that the increase of temperature is conducive to forming the α phase, thus causing the temperature to increase and F(β) to weaken. Huang et al. studied the F(β) of nanofibers at different ambient temperatures (5, 15, 25, 35, and 45 °C) [131]. As the temperature increases, F(β) increases, PVDF nanofibers get the maximum F(β) at 25 °C. The F(β) decreases slightly, when the temperature continues to increase. To further analyze the experimental results, the authors tested the PVDF solution’s surface tension and viscosity at different temperatures. The results show that as the temperature increases from 5 to 45 °C, the PVDF solution’s surface tension decreases from 41.23 to 27.05 mN/m and the viscosity decreases from 241.8 to 112.6 cP. This causes higher elongation forces to be imposed on jets. Then, the diameter of the nanofibers is reduced, and the F(β) value is increased. However, an excessively high temperature is not conducive to forming the β phase, so room temperature (25 °C) is currently used as the ambient temperature for electrospinning [139,140,141].

## 5. Introduction of Nanoparticles to the Structure of Nanofibers for Improving the β Phase

In addition to the process parameters, many researchers have used nanoparticles as additives to a polymer solution that ultimately affect the β phase of PVDF nanofibers. These nanoparticles are mainly divided into organic and inorganic nanoparticles. Table 2 lists some commonly used nanoparticles.

### 5.1. Inorganic Nanomaterials

The addition of inorganic nanomaterials to a polymer solution via electrospinning has been used in many experiments, and there are many types of compounds used, such as lithium chloride (LiCl) [92], silver nanowires (AgNWs) [142], zinc oxyde (ZnO) [143], potassium sodium niobite (KNN) [145], and barium titanate (BaTiO3) [144].

In order to achieve change in the conductivity of a PVDF solution prepared with the same solvent, inorganic salt is generally used. Mokhtari et al. added LiCl to a 16 wt % PVDF/DMF/acetone solution [92]. The results showed that although excess LiCl causes the solution to produce bending instability under an electric field, adding an appropriate amount of inorganic LiCl salt could increase the solution’s conductivity, thereby effectively reducing the formation of the bead structure. The change in morphology of electrospinning fibers can be attributed to the increase in the polymer solution’s charge density because of the improvement of the conductivity [149]. On the other hand, with the local dipole field on the inorganic nanoparticles’ surface with metallic characteristics, the PVDF molecular chain could be promoted to show the all-trans (TTTT) conformation under an electric field. Compared with PVDF electrospinning nanofibers without AgNWs, in the XRD spectra patterns of PVDF nanofibers with 1.5% AgNWs, the α phase intensity diffraction peak almost disappears [142]. AgNWs are nanoparticles with metallic properties, an electron-rich material with a local dipole field on the surface, which promotes the polymer chain to exhibit a TTTT conformation. Then, the PVDF chains form a polar β phase on the surface of nanoparticles.

The intense interaction between inorganic nanomaterials and PVDF leads to an increase in F(β). For example, KNN, as an α-nucleating agent, is certainly not conducive to the formation of the PVDF β phase. However, in the Bairagi et al.’s experiment, it was found that although the addition of 1 wt % KNN reduces the F(β) of PVDF from 97% to 61% when the content of KNN increases to 5 wt%, F(β) increases to 95% [145]. They attributed this result to the strong interaction between KNN and the PVDF molecular chain. Even if the additive is an α-nucleating agent, the β phase can still be improved when the interaction between the additive and the molecular chain is strong.

### 5.2. Organic Nanomaterials

Organic nanomaterials are used as additives to improve the β phase of PVDF nanofibers in many researches, including graphene [85], carbon nanotubes (CNTs) [105], tetramethylammoniumchloride (TMAC) [118], tetrabutyl-ammonium chloride (TBAC) [64,150], 2,3-dihydrodecafluoropentane [82], and 1-octadecyl-3-methylimidazolium bromide ([OMD]Br) [97]. Organic nanoparticles can enhance the β phase of PVDF nanofibers, attributed to the strong interaction between organic nanomaterials and PVDF. Xing et al. used 1-butyl-3-methylimidazolium hexafluorophosphate ([BMIM][PF6]) as an additive and the interaction between the additive and PVDF [148]. With the interaction of imidazolium ions of the additives with CF_2_, the TTTT configuration of PVDF is further stabilized, so a large amount of β crystal is obtained. Simultaneously, the authors also compared PVDF nanofibers with [BMIM][PF6] added to pure PVDF nanofibers and found that although pure PVDF nanofibers are still dominated by the β phase, there is a small amount of the γ phase. For PVDF nanofibers with [BMIM][PF6], there is almost the 100% β phase without the γ phase. This is because in the electrospinning process, the PVDF just ejected was still in the state of solution. Under the high energy provided by an electric field, the unstable TTTT configuration of the β phase is converted to the TTTG configuration of the γ phase. Due to the strong interaction between [BMIM][PF6] and PVDF, the unstable β phase configuration of PVDF is stabilized, thereby preventing the β phase’s conversion to the γ phase.

Mahdavi et al. added 0.25, 1, and 4 wt % of 1-octadecyl-3-methylimidazolium Bromide ([OMD]Br) to 22 wt % of a PVDF/DMF/acetone solution [97]. The results showed that the F(β) of pure PVDF nanofibers is 76.04%. After adding [OMD]Br, the F(β) of PVDF nanofibers is greater than 95%, and F(β) increases with the addition of [OMD]Br. When the content of [OMD]Br is 4 wt %, the F(β) of PVDF nanofibers is 98.62%. There are several interactions in the PVDF/DMF/acetone/[OMD]Br solution, i.e., the interactions between the positive charges of [OMD]Br and the fluorine atoms in PVDF and the interactions between negative charges of [OMD]Br and the hydrogen atoms in PVDF. A local electric field is formed at the ionic bond location, which results in the dipole alignment and creates the β phase in PVDF.

CNTs have a strong interaction with PVDF molecular chains and are widely used as nanoparticles in PVDF electrospinning [147]. Although CNTs are randomly dispersed in an electrospinning solution, they are arranged along the flow direction at a Taylor cone [139]. In electrospinning, PVDF molecular chains and CNTs are aligned along with the polarization and stretching directions. Due to the strong interaction between CNTs and PVDF molecular chains, PVDF molecular chains and SWCNTs are aligned along the fiber axis and would not be destroyed, conducive to the formation of highly oriented β-type crystals at the interface [151]. The enhancement effect of CNTs on the β phase in PVDF nanofibers has been confirmed in many experiments. Sharma et al. added 1 wt % of CNTs to a 25 wt % of PVDF/DMF/acetone solution, leading to an increase of F(β) from 78% of pure PVDF to 84% [67]. Adding 0.2 wt % of MWCNTs increases the F(β) values of PVDF nanofibers from 80% to 90% [139].

In addition to the interaction between additives and PVDF, hydrogen bonds are also generated to act on the PVDF molecular chain to stabilize and improve the β phase of PVDF. Nasir et al. added 6 wt % of 2,3-dihydrodecafluoropentane to a PVDF/DMAc solution for electrospinning. In this solution, the positive charges of PVDF (hydrogen atoms) interact with the additive (fluorine atoms) negative charges [82]. The hydrogen atoms of the additive and the PVDF fluorine atoms also form hydrogen bonds. Simultaneously, the fluorine atoms of the additive and the PVDF hydrogen atoms also form a hydrogen bond. The hydrogen bond between the additive and the β-phase PVDF is closer and more favorable than the hydrogen bond between the additive and the α-phase PVDF. Under the applied voltage, the strong hydrogen bond interaction makes the α phase change to the β phase, increasing the β phase of PVDF nanofibers.

## 6. Piezoelectric Properties on Nanofibrous Membranes

The commonly used test method is piezoelectric force microscopy (PFM) to investigate piezoelectric properties on nanofibrous membranes. The tip of the PFM is fixed on an electrospinning fiber, and a voltage is applied to the fiber. The voltage causes the deformation of the internal structure of the fiber and is detected by the tip. The images of the amplitude and phase obtained by the piezoelectric reaction reflect the piezoresponse magnitude and polarization direction within the fibers. The hysteresis loop obtained under voltages provides information about the ferroelectric properties of the fibers. The maximum amplitude of the fiber can be obtained by PFM amplitude and DC voltage hysteresis loops.

**Figure 4:**
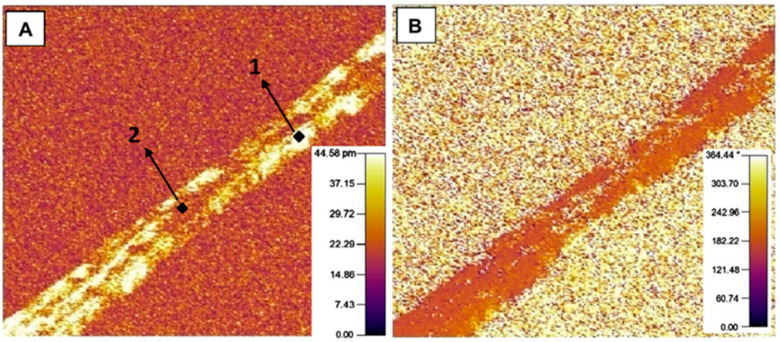
(**A**) Piezoelectric force microscopy (PFM) amplitude image. (**B**) PFM phase image [152]. The sample size is 4 μm × 4 μm (Two points on the fiber refers to those selected by the authors to measure the piezoresponse at selected localized regions. Point 1 is located at a region which shows a bright contrast while Point 2 is at a region which has a dark contrast in the PFM amplitude image).

Baji et al. used PMF to determine the nanofibers’ piezoelectric properties (Figure 4) [152]. The piezoresponse magnitude can be estimated from an amplitude image. In the phase image, a bright central region and a dark region can be seen. Therefore, the central region of the composite fiber has the opposite polarization compared to the dark region.

Gebrekrstos et al. have determined the maximum amplitude of three nanofibers PVDF/GO, PVDF/GOCOOH, and PVDF/GOF by PFM [153]. The d33 depends on the β phase content of PVDF nanofibers and is found to be increased with the increase of the β content. In the Szewczyk et al.’s study, electrospinning was performed at 30% and 60% RH, and the voltage was 15 kV with positive and negative polarity [135]. They observed that when the β phase content is the highest, the obtained d33 is the largest.

In addition to PFM, three other types of methods are also used to measure the piezoelectric properties of nanofibrous membranes, including frequency, laser interferometry, and quasi-static methods [154]. Soraya Bafqi et al. used the frequency method on PVDF/ZnO nanofibrous membranes [143]. A criterion piezoelectric layer was placed on a PVDF nanofibrous membrane, and oil was added between the criterion piezoelectric layer and the sample to get better contact. The voltage and frequency (21 V, 75 KHz) provided by a function generator caused the vibrations of the criterion piezoelectric layer and the sample because of the converse piezoelectric effect. Due to the influence of piezoelectricity, the electrical responses generated by these vibrations were measured by an oscilloscope to obtain the voltage output. Through this method, the piezoelectric properties of the sample can be effectively measured. When the ZnO content is 15%, the sample’s output voltage is the highest, increasing from 315 to 1100mV. When the ZnO content increases from 0% to 15%, F(β) increases from 80% to 87% and Xc increased from 51.4% to 54.5%. This result shows that the content of the β phase in PVDF directly affects the piezoelectric properties of PVDF nanofibrous membranes.

Another method of measuring piezoelectric properties is the laser interferometry method. Fabriani et al. have used this method to measure PVDF nanofibrous membranes’ piezoelectric properties [155]. The PVDF nanofibrous membranes were installed between the two electrodes and connected to an oscilloscope, which were then placed on a fiberglass beam. On releasing the fiberglass beam, oscillations were produced. A laser vibrometer detected these oscillations: the laser recorded the relationship between speed and time, and the oscilloscope recorded the relationship between voltage and time. The results showed that these two curves are almost coincident. This illustrates the feasibility of the laser interferometry method to measure the piezoelectric properties of samples. However, the laser interferometry method requires high experimental accuracy, and any slight irregular vibration during the measurement process would affect the accuracy of the results.

Compared with the frequency method and the laser interferometry method, the quasi-static method does not require such high experimental accuracy, and the equipment is simple and easy to set up at low cost. Therefore, among the current methods for measuring PVDF nanofiber membranes’ piezoelectric properties, the quasi-static method is very common. The principle is to deform a static nanofiber membrane through a specific method (pressure, displacement, stretching, etc.) and then measure the output voltage change through an oscilloscope as a function of the piezoelectric response.

Lee et al. have placed their sample (PVDF/BaTiO_3_ fibrous bundle) on two stages with an interval of 1 mm and connected electrodes at both ends to measure the voltage generated by piezoelectricity [127]. One stage is static, and the other stage is mobile. By moving another stage, the sample was deformed, and a voltage was generated. The test frequency can be changed by changing the speed of the mobile stage. Al Halabi et al. have used gravity to deform PVDF nanofibrous membranes and measured the samples’ piezoelectric properties [156]. The sample was compressed by gravity to measure the voltage difference on the surface when a weight was placed.

Some quasi-static method equipment deforms the membranes by changing the pressure, thereby testing the membrane’s piezoelectric properties [157,158], and includes different devices such as gas sources, pressure and frequency control units, signal generators, DC power suppliers, PVDF sensors, and oscilloscopes. By adjusting the gas flow rates, the gas passes through a PVDF sensor at a determined pressure and frequency, which causes the deformation of the PVDF nanofibrous membrane in the PVDF sensor. On the one hand, the results proved that the PVDF nanofibrous membranes prepared by electrospinning have good piezoelectric properties (0.6 V) because of the rich β phase content. On the other hand, although the frequency change has little effect on the output voltage, the output voltage shows noticeable pressure sensitivity (178 mV·kPa^−1^) [157].

## 7. Conclusions

Due to its high piezoelectric constants, flexibility, the variability of the manufacturing processing, and the moderate cost compared to high specialty copolymers, PVDF has garnered research interest for their potential use and advantages in applications as energy harvesters, sensors, etc. To maximize the PVDF’s piezoelectric performances, the total polar phase contents (β and γ phases) of this polymorph polymer need to be enhanced. A current solution to reach this objective is to use the electrospinning process and to develop nanofibers. By applying a strong electrical potential between a polymer and a collector, a jet of the polymer under nanofibers is obtained with a simple process called solution or melt electrospinning. Three significant forces, i.e., the viscoelastic force, the electric force, and the surface tension, govern the electrospinning process, and three major parameters (properties linked to the polymer and/or to the solution, controlled variables of the process, and the ambient parameters) tailor the electrospun nanofibers. Solution electrospinning is often preferred to melt spinning, because it requires working with higher electric fields and with polymers with specific viscosities. In the solution electrospinning, the preparation of the solution is one of the keys to obtaining nanofibers with desired properties. A suitable solvent with adequate properties (surface tension, vapor pressure, dielectric constant, boiling point, etc.) as well as the selection of PVDF (the molecular weight and molecular weight distribution) and the concentrations of these two components needs to be considered carefully. As all these parameters interact, it is complex to find a consensus to ensure the best stretchability of a PVDF jet leads to the highest β phase content. The process parameters also need to be adjusted very carefully, as they affect the total polar phase content of the PVDF and ultimately its piezoelectric performance. Indeed, depending on the chosen voltage or even the flow rate, the stretching of the polymer jet is modified and consequently the PVDF crystalline structure (crystallinities, types, and contents of different crystalline phases).

Furthermore, some studies pointed out that RH or ambient temperature could also affect the formation of the β phase of PVDF. In addition, in order to optimize the piezoelectric response of PVDF, some research teams have been interested in new ways by modifying some aspects of the electrospinning process (collector, etc.) or investigating the influence of additives (organic or inorganic nanoparticles) on the PVDF crystalline structure. Besides the difficulty of choosing the right set of elements to obtain electrospun PVDF nanofibers with the highest beta phase content, the piezoelectric properties’ measurements are still complex and primarily performed by using PFM.

## Figures and Tables

**Table 1:** Summary of different collectors in electrospinning.

Type of Collector	Advantages	Disadvantages	References
Static collector 1. Metal plate collector 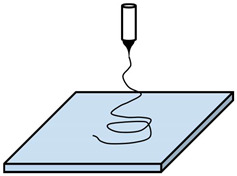	Simple equipment	Random distribution of nanofibers Not conducive to stretching the nanofibers Low F(β)	[91,92]
2. Double-plate collector 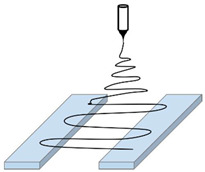	Highly aligned nanofibers Enhance F(β) (compared with a mental plate collector)		[93,94,]
3. Circle electrode collector 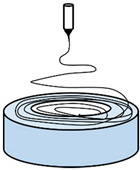	Easy to transfer membranes to the target substrates High productivity	Random distribution of nanofibers Low F(β)	[95]
Rotating collector 1. Rotating drum collector 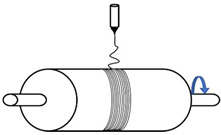	Highly aligned nanofibers Enhance F(β) (compared with a mental plate collector) The large area of a nanofibrous membrane		[96,97]
2. Rotating disk collector 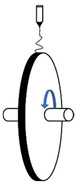	Highly aligned nanofibers Enhance F(β) (compared with all other collectors)	The small area of a nanofibrous membrane	[98,99,100]

**Table 2:** Common nanomaterials used to improve the β phase content.

	Name of Nanomaterials	How to Improve the β Phase	Reference
Inorganic nanomaterials	LiCl	Improvement of the solution conductivity	[92]
	AgNWs	Improvement of the solution conductivity	[142]
	ZnO	Improvement of the solution conductivity	[143]
	BaTiO_3_	Local dipole field on the surface	[144]
	KNN	Beta-nucleating agent in PVDF	[145]
Organic nanomaterials	Graphene	Electro-interactions	[82,146]
	CNTs	Interfacial polarization	[85]
	[BMIM][PF6]	Strong interactions	[97]
	[OMD]Br	Strong interactions	[147]
	2,3-dihydrodecafluoropentane	Hydrogen bonds	[148]

## Data Availability

No new data were created or analyzed in this study. Data sharing is not applicable to this article.
